# Effects of regional cerebral oxygen saturation monitoring on postoperative cognitive dysfunction in older patients: a systematic review and meta-analysis

**DOI:** 10.1186/s12877-023-03804-6

**Published:** 2023-03-06

**Authors:** Xiahao Ding, Tianming Zha, Gulibositan Abudurousuli, Cuimei Zhao, Zixuan Chen, Yang Zhang, Bo Gui

**Affiliations:** 1 Department of Anesthesiology and Perioperative Medicine, 1st Affiliated Hospital with Nanjing Medical University, No. 300 Guangzhou Road, 210029 Nanjing, Jiangsu China; 2 Department of Anesthesiology, Nanjing Qixia District Hospital, 210046 Nanjing, China

**Keywords:** Older patients, Meta-analysis, Postoperative cognitive dysfunction, Regional cerebral oxygen saturation

## Abstract

**Background:**

Postoperative cognitive dysfunction (POCD) is common after surgery and anesthesia, particularly in older patients. It has been reported that regional cerebral oxygen saturation (rSO_2_) monitoring potentially influences the occurrence of POCD. However, its role in the prevention of POCD remains controversial in older patients. Additionally, the quality of evidence on this topic is still relatively poor.

**Methods:**

The electronic databases PubMed, EMBASE, Web of Science, and Cochrane Library were systematically searched using the indicated keywords from their inception to June 10, 2022. We limited our meta-analysis to randomized controlled trials (RCTs) that assessed the effects of rSO_2_ monitoring on POCD in older patients. Methodological quality and risk of bias were assessed. The primary outcome was the incidence of POCD during hospitalization. The secondary outcomes were postoperative complications and the length of hospital stay (LOS). Odds ratios (OR) and 95% confidence intervals (CI) were calculated to determine the incidence of POCD and postoperative complications. The standardized mean difference (SMD) instead of the raw mean difference and 95% CI were calculated for LOS.

**Results:**

Six RCTs, involving 377 older patients, were included in this meta-analysis. The incidence of POCD ranges from 17 to 89%, with an overall prevalence of 47% in our pooled analysis. Our results demonstrated that rSO_2_-guided intervention could reduce the incidence of POCD in older patients undergoing non-cardiac surgery (OR, 0.44; 95% CI, 0.25 to 0.79; *P* = 0.006) rather than cardiac surgery (OR, 0.69; 95% CI, 0.32 to 1.52; *P* = 0.36). Intraoperative rSO_2_ monitoring was also associated with a significantly shorter LOS in older patients undergoing non-cardiac surgery (SMD, -0.93; 95% CI, -1.75 to -0.11; *P* = 0.03). Neither the incidence of postoperative cardiovascular (OR, 1.12; 95% CI, 0.40 to 3.17; *P* = 0.83) nor surgical (OR, 0.78; 95% CI, 0.35 to 1.75; *P* = 0.54) complications were affected by the use of rSO_2_ monitoring.

**Conclusion:**

The use of rSO_2_ monitoring is associated with a lower risk of POCD and a shorter LOS in older patients undergoing non-cardiac surgery. This may have the potential to prevent POCD in high-risk populations. Further large RCTs are still warranted to support these preliminary findings.

**Supplementary Information:**

The online version contains supplementary material available at 10.1186/s12877-023-03804-6.

## Background

With the advancement of medical technology, an increasing number of older patients have gained access to surgical treatment in recent decades. To date, approximately half of the older population is estimated to have undergone at least one surgery. Compared to younger patients, the older population has been reported to be much more prone to developing perioperative complications following surgery and anesthesia due to a higher prevalence of comorbidities and increased perioperative risk [1, 2]. Postoperative cognitive dysfunction (POCD), a common complication of surgery and anesthesia, has been recognized as a new-onset cognitive impairment after any type of surgical intervention, including cardiac and non-cardiac surgery, especially in the geriatric population, which may persist for a few days, months, or even years [3, 4]. POCD is broadly characterized by a series of alterations in neurocognitive conditions and behavior, including impaired memory, poor comprehension, and reduced attention [5]. Previous studies have revealed that the incidence of POCD in older individuals varies from 16.7 to 89% one week after surgery [6–9]. It is well established that POCD is associated with a range of negative outcomes, including prolonged hospitalization, changes in mood and personality, reduced quality of life, heavy burden on the family and society, and increased mortality [10, 11].

Regional cerebral oxygen saturation (rSO_2_) monitoring, a non-invasive method to monitor cerebral perfusion and ischemia that is measured by near-infrared spectroscopy (NIRS), has played an essential role in guiding or optimizing perioperative management [12]. Several studies have revealed that a lower level of rSO_2_ during surgery is strongly associated with an increased risk of POCD [13–15]. However, other studies failed to reveal the potential association between intraoperative rSO_2_ values and the incidence of POCD [16–18], which raises the question of the clinical validity of rSO_2_ monitoring in preventing POCD, especially in older surgical patients. Furthermore, no consensus exists regarding the role of rSO_2_ monitoring-based management in the prevention of POCD. Additionally, the quality of evidence on this topic is relatively poor among currently published meta-analyses [19–22]. Moreover, the clinical value of rSO_2_ between cardiac and non-cardiac surgical patients has not yet been established.

Considering that the quality of life among older patients is impaired by long-standing POCD and that the conflicting results vary from existing studies, we conducted this systematic review and meta-analysis to gather the existing literature and explore the association between rSO_2_ monitoring and early POCD during hospitalization in older patients. We hypothesized that rSO_2_-based perioperative management has a predictive value for the incidence of POCD and other common adverse events in this high-risk population.

## Methods

This meta-analysis was conducted in accordance with the recommendations and guidelines of the Cochrane Handbook for Systematic Reviews of Interventions (Version 6.3). A checklist is included in **Additional file 1**. The protocol was registered in the International Prospective Register of Systematic Reviews (PROSPERO) at the National Institute for Health Research (CRD42020204570). As the present analysis was based on a systematic review of previously published studies, institutional review board approval and patient consent were deemed unnecessary.

## Search strategy

A systematic literature search was conducted by one reviewer (X.H.D.) on PubMed, EMBASE, Web of Sciences, and Cochrane Library from the inception of each database to June 10, 2022. We used a combination of Medical Subject Headings (MeSH) and keywords with various synonyms that reflect the following concepts: “POCD,” “rSO_2_” and “older”. We also manually examined the reference lists of relevant articles to identify other eligible sources. No language restriction was applied and no filter for publication type were used. Full details of the search strategy, including the complete search strings, are available in **Additional file 2**.

## Study selection criteria

We limited our meta-analysis to randomized controlled trials (RCTs) assessing the effects of rSO_2_ monitoring on POCD. We also selected studies based on the following criteria: (a) participants aged ≥ 60 years; (b) the study had at least one intervention group (rSO_2_-guided anesthesia) and one control group (routine care); (c) patients who had surgeries under anesthesia; and (d) the occurrence of POCD evaluated by definitive diagnostic criteria was reported in the study. Studies were excluded if they were: (a) not an RCT; (b) ongoing studies; (c) not full-text studies; (d) duplicates of previous reports; and (e) unable to extract data for analysis.

Two reviewers (G.A. and C.M.Z.) independently and in parallel screened the titles and abstracts. The full texts of the selected articles were then retrieved and screened for eligibility by the same reviewers. In cases of disagreement between the reviewers, a consensus was ultimately reached within the author group for a final decision.

## Data extraction

Using standardized forms, two reviewers (Y.Z. and Z.X.C) independently extracted relevant information. After independent data extraction, the two reviewers consulted each other to identify disagreements and reach a consensus with the third reviewer (X.H.D.). The data extraction form included the following: first author, publication year, participants, sample size of each group, type of surgery, intervention, monitoring device, assessment methods for POCD, definition of abnormal rSO_2_ values, and outcomes measured. We contacted the authors of eligible studies if missing data associated with the analysis were pertinent to our analysis.

## Assessment of methodological quality and risk of bias

Two reviewers (G.A. and T.M.Z.) independently assessed the risk of bias of individual studies according to version 2 of the Cochrane tool for assessing the risk of bias in randomized trials (RoB2) [23]. Any disagreements were resolved by group discussion or by a third reviewer (B.G.). For each study, we assessed the risk of bias in the following domains: randomization process, deviations from intended interventions, missing outcome data, measurement of the outcome, and selection of the reported result. We judged each study as having a low or high risk of bias, or some concerns with respect to the level of risk of bias.

The methodological quality of outcomes pooled across trials was independently evaluated using the Grades of Recommendation, Assessment, Development, and Evaluation (GRADE) by the two reviewers [24]. The quality of evidence for each outcome was graded as high, moderate, low, or very low based on the following domains: risk of bias, inconsistency, imprecision, indirectness, and publication bias. Studies with both high and moderate quality are referred to as high quality, whereas low and very low graded studies are considered low quality.

## Primary and secondary outcomes

The primary outcome of interest was the incidence of POCD ascertained seven days after surgery or, if not reported, the time point closest to seven days during hospitalization after surgery. Neuropsychological tests for the diagnosis of POCD were performed before and within one week after surgery. The secondary outcomes included postoperative complications and length of hospital stay (LOS).

## Statistical analysis

All statistical analyses were performed using Review Manager (version 5.3; Cochrane Collaboration, Oxford, UK) and R software (version 4.2.0; The R Foundation, Vienna, Austria). Odds ratios (OR) and 95% confidence intervals (CI) were calculated using the Mantel-Haenszel method for dichotomous outcomes (e.g., the incidence of POCD and postoperative complications). The standardized mean difference (SMD), instead of the raw mean difference, and 95% CI were calculated using the inverse variance method for continuous variables (e.g., LOS). Statistical heterogeneity across studies was assessed using *I*^*2*^ and Cochran’s *Q* test values, where an *I*^*2*^ value of more than 50% and a Cochran’s *Q* test with a *P* < 0.10 was considered significant for heterogeneity [25]. If *I*^*2*^ was > 50% or *P* < 0.10, the random effects model was used because of considerable heterogeneity among the studies. Otherwise, the fixed-effects model was applied. Subgroup analyses based on the type of surgery (cardiac vs. non-cardiac surgery) were subsequently performed to obtain more specific results. L’Abbe, Galbraith, and Baujat plots were constructed to explore the relative contribution of each primary-level study to the overall heterogeneity in more depth and to control for the presence of potential outliers. A funnel plot was established to determine the existence of potential publication bias by visual inspection of the asymmetry. Additionally, we performed Egger’s tests to evaluate publication bias among the included studies, although its capacity to detect such bias was limited when meta-analyses were based on a limited number of small trials [26]. A significant publication bias was considered when there was asymmetry in the former, and a statistically significant bias coefficient was noted in the latter. *P* < 0.05 was considered statistically significant for all the statistical tests.

## Results

### Literature search

**Fig. 1:**
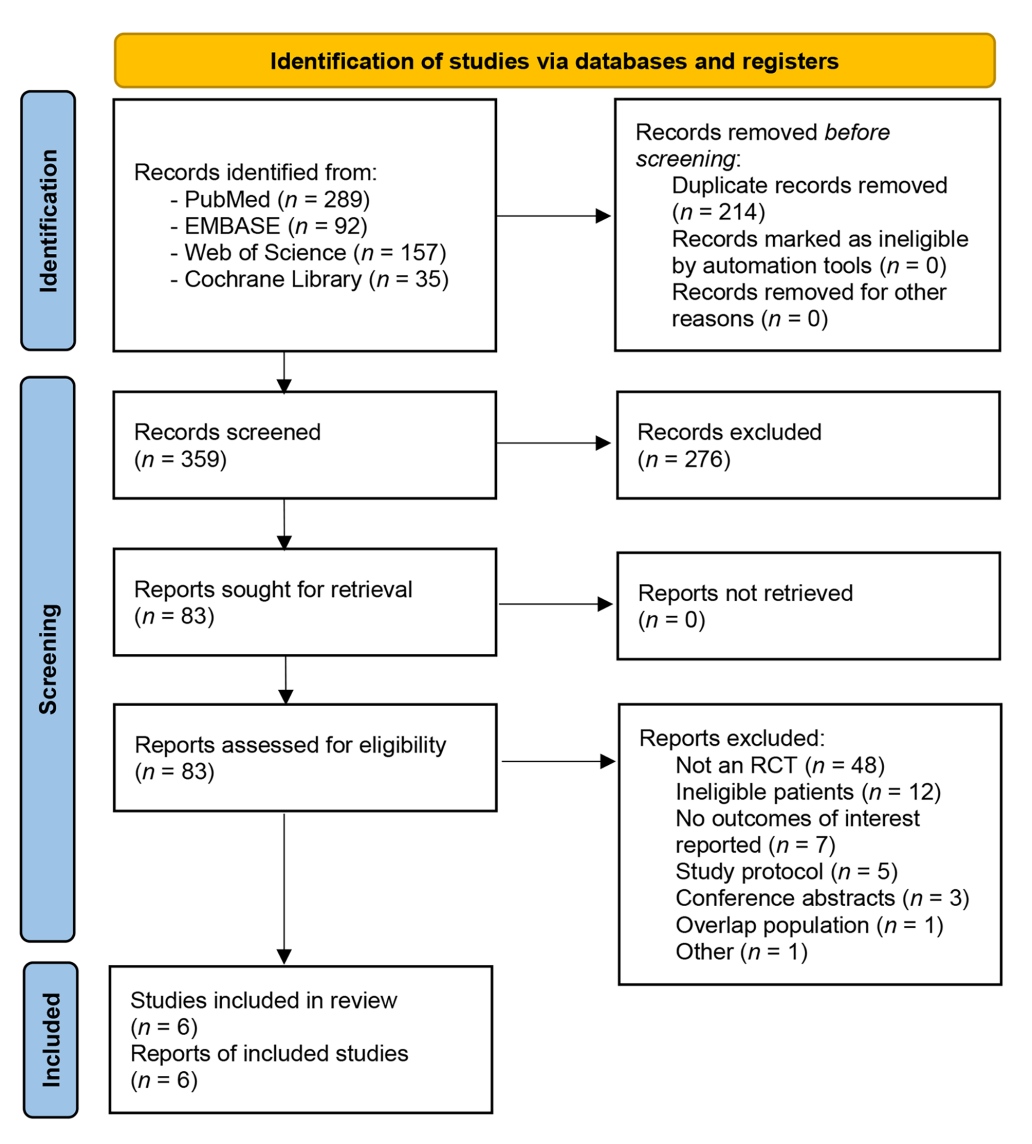
The flowchart for study screening and selection process

A flowchart of the study screening and selection process is shown in Fig. 1. A total of 573 relevant publications were identified. After removing 214 duplicate studies, 359 studies were screened at the title and abstract levels. A total of 276 studies were excluded because they failed to meet our inclusion criteria. Of the remaining 83 eligible studies, we further excluded a total of 77 articles for one or more of the following reasons: not an RCT (*n* = 48), ineligible participants under the age of 60 (*n* = 12), no outcomes of interest reported (*n* = 7), study protocol (*n* = 5), conference abstracts with data that could not be extracted for analysis (*n* = 3), overlap population (*n* = 1), and irrelevant study involving non-general anesthesia patients (*n* = 1). Ultimately, six RCTs involving 377 older patients were included in this meta-analysis [6, 7, 27–30].

### Characteristics of included studies

The detailed clinical characteristics of the included studies are summarized in Table 1. Among these RCTs, two included 122 older patients who underwent cardiac surgery [29, 30], whereas the other four studies included 255 older patients who underwent non-cardiac major surgery, including abdominal non-vascular surgery and orthopedic surgery [6, 7, 27, 28]. All patients were randomly divided into intervention and control groups. In the intervention group, patients were managed with continuous monitoring of rSO_2_ using NIRS and treated with several interventions to maintain the rSO_2_ value within a certain range according to their own definition. In the control group, patients were treated with routine anesthesia management without monitoring of rSO_2_ or visualization of perioperative rSO_2_ values. Indications for the correction of cerebral desaturation included a drop of more than 15% [29], 20% [7, 28, 30] or 25% [27] from baseline rSO_2_ values and/or any absolute rSO_2_ value less than 50% [6, 28–30]. The intervention for optimization of the rSO_2_ value included alternation of the patient’s head position, correction of the pressure of arterial carbon dioxide (PaCO_2_) value or fractional percentage of inspired oxygen (FiO_2_), administration of vasoactive drugs, or red blood cell transfusion. The sample size ranged from 26 to 122 and the mean age of the participants ranged from 64.8 to 75.7 years. The majority of included studies used the Mini-Mental State Examination (MMSE) to assess cognitive function [6, 27–30], two studies used the Montreal Cognitive Assessment (MoCA) [7, 28], and one study used a comprehensive neuropsychological test battery [30]. All patients were assessed before surgery for baseline cognitive evaluation and then repeated during the postoperative hospital stay, such as 1–3 [28], 3–5 [29], or 7 days [6, 7, 27, 30].

**Table 1:** Characteristics of studies included in the analysis

Study	Sample size	Groups	Participants	NIRS device	Surgery type	Assessment methods	Assessment timeline	Definition of cerebral desaturation	Quality of the evidence (GRADE)
Yang et al., 2021 [7]	26	IG (*n* = 12) CG (*n* = 14)	> 65 yr	Masimo	spinal surgery	MoCA	pre-surgery, postoperative 7 d	a drop of more than 20% from baseline value	⊕⊕⊕⊕ High
Jing et al., 2021 [28]	60	IG (*n* = 30) CG (*n* = 30)	> 60 yr	OXImeter	shoulder arthroscopic surgery	MMSE, MoCA	pre-surgery, postoperative 1–3 d	a drop of more than 20% from baseline value or rSO_2_ less than 50%	⊕⊕⊕○ Moderate
Kunst et al., 2020 [29]	82	IG (*n* = 42) CG (*n* = 40)	> 65 yr	INVOS	CABG	MMSE	pre-surgery, postoperative 3–5 d	a drop of more than 15% from baseline value or rSO_2_ less than 50%	⊕⊕⊕⊕ High
Şahan et al., 2018 [30]	40	IG (*n* = 19) CG (*n* = 21)	> 60 yr	INVOS 5100 C	CABG	MMSE, WMS, CDT, WLGT, DSS, VSST	pre-surgery, postoperative 7 d, postoperative 3 m	a drop of more than 20% from baseline value or rSO_2_ less than 50%	⊕⊕⊕⊕ High
Ballard et al., 2012 [6]	47	IG (*n* = 19) CG (*n* = 28)	> 60 yr	INVOS	Orthopedic, abdominal surgery	MMSE	pre-surgery, postoperative 7 d	a drop of more than 15% from baseline value or rSO_2_ less than 50%	⊕⊕○○ Low
Casati et al., 2005 [27]	122	IG (*n* = 56) CG (*n* = 66)	> 65 yr	INVOS 4100	major abdominal, nonvascular surgery	MMSE	pre-surgery, postoperative 7 d	a drop of more than 25% from baseline value (20% in case of baseline rSO_2_ < 50%) for ≥ 15 s.	⊕⊕⊕○ Moderate

CABG, coronary artery bypass graft; CDT, clock drawing test; CG, control group (routine care); DSS, digit span subtest; GRADE, Grading of Recommendations Assessment, Development and Evaluation; IG, intervention group (rSO_2_-guided); MMSE, mini-mental state exam; MoCA, Montreal cognitive assessment; NIRS, near-infrared spectroscopy; rSO_2_, regional cerebral oxygen saturation; VSST, visuo-spatial skills test; WLGT, word list generation test; WMS, Wechler memory scale

### Quality assessment and risk of bias

**Fig. 2:**
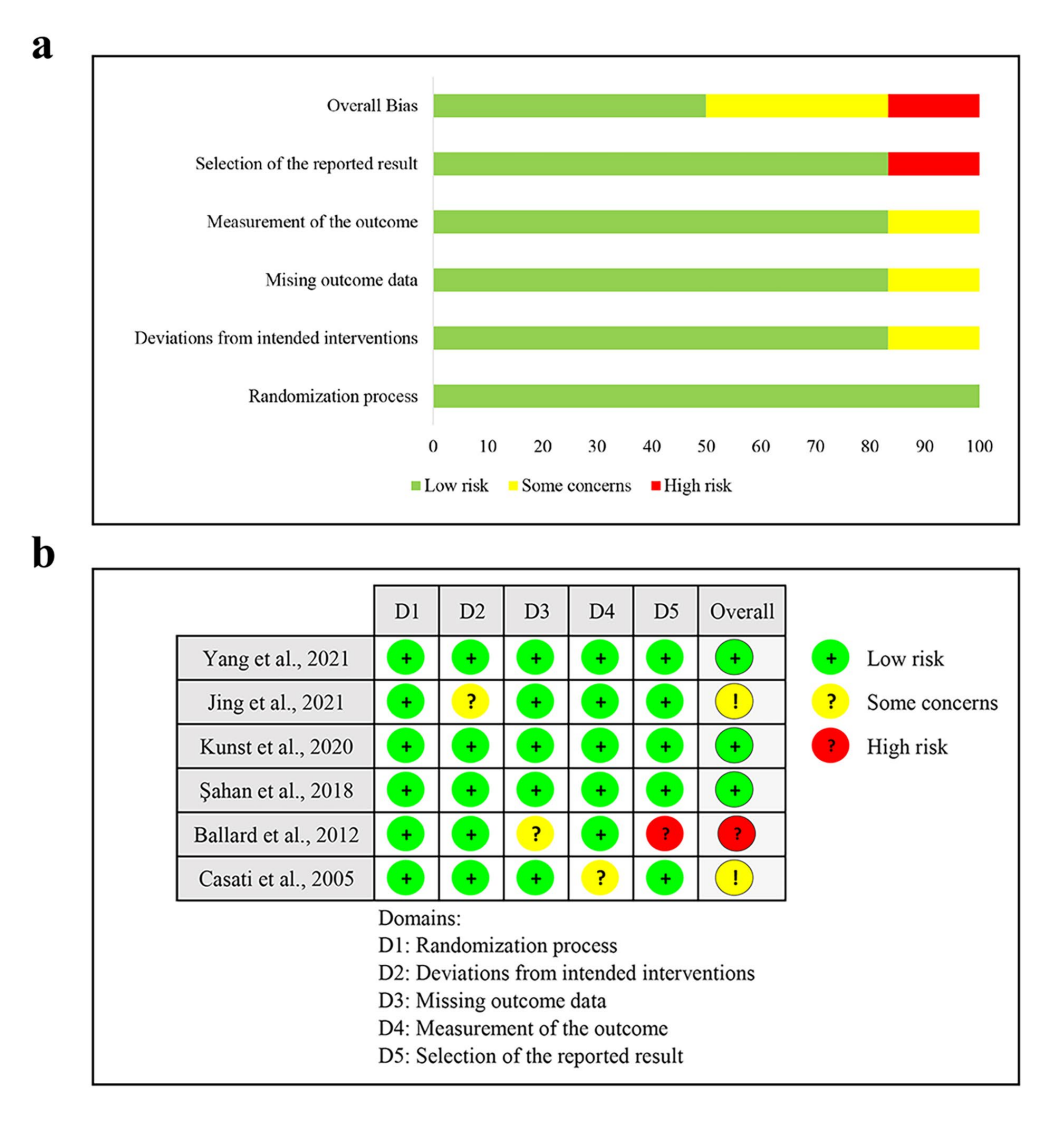
Assessment of risk of bias based on the version 2 of the Cochrane tool for assessing the risk of bias in randomized trials (RoB2). **(a)** Percent of studies with categories for risk of bias; **(b)** Summary for the risk of bias in each study

The quality and risk of bias of the included studies were assessed and the results are shown in Fig. 2. One study was judged to have a high risk of bias in the selection of the reported results [6]. Two studies had some concerns due to bias in the deviations from the intended interventions or measurement of the outcome [27, 28]. Generally, the majority of the included studies were assessed as having a low risk of bias, which indicated that they were of moderate-to-high quality.

### Postoperative cognitive dysfunction (POCD)

**Fig. 3:**
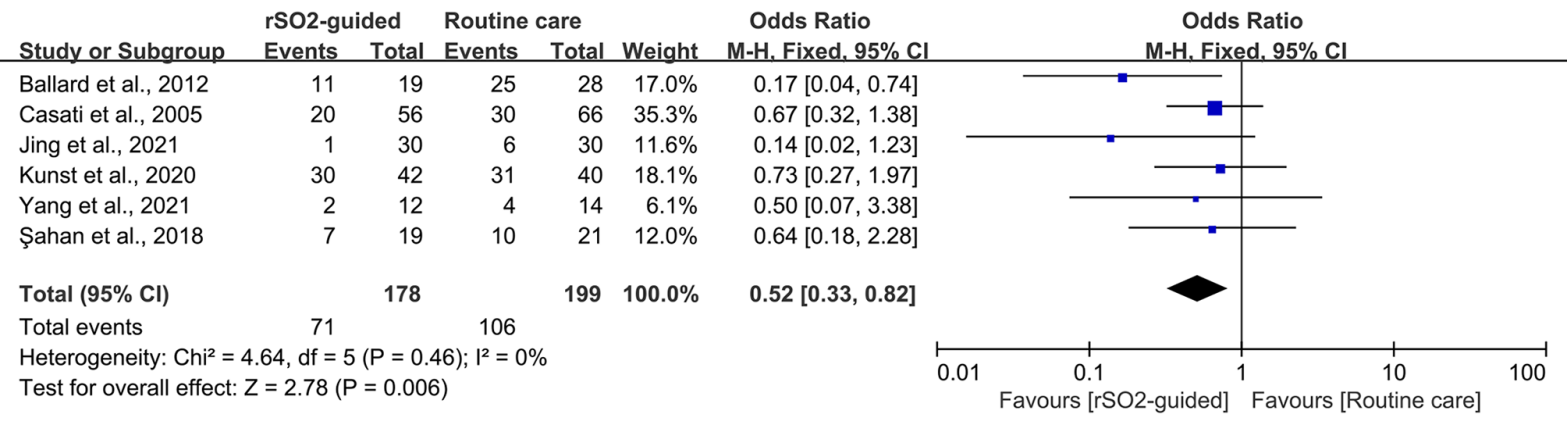
Forest plot illustrating the incidence of POCD between intervention group (rSO_2_-guided anesthesia) and control group (routine care)

**Fig. 4:**
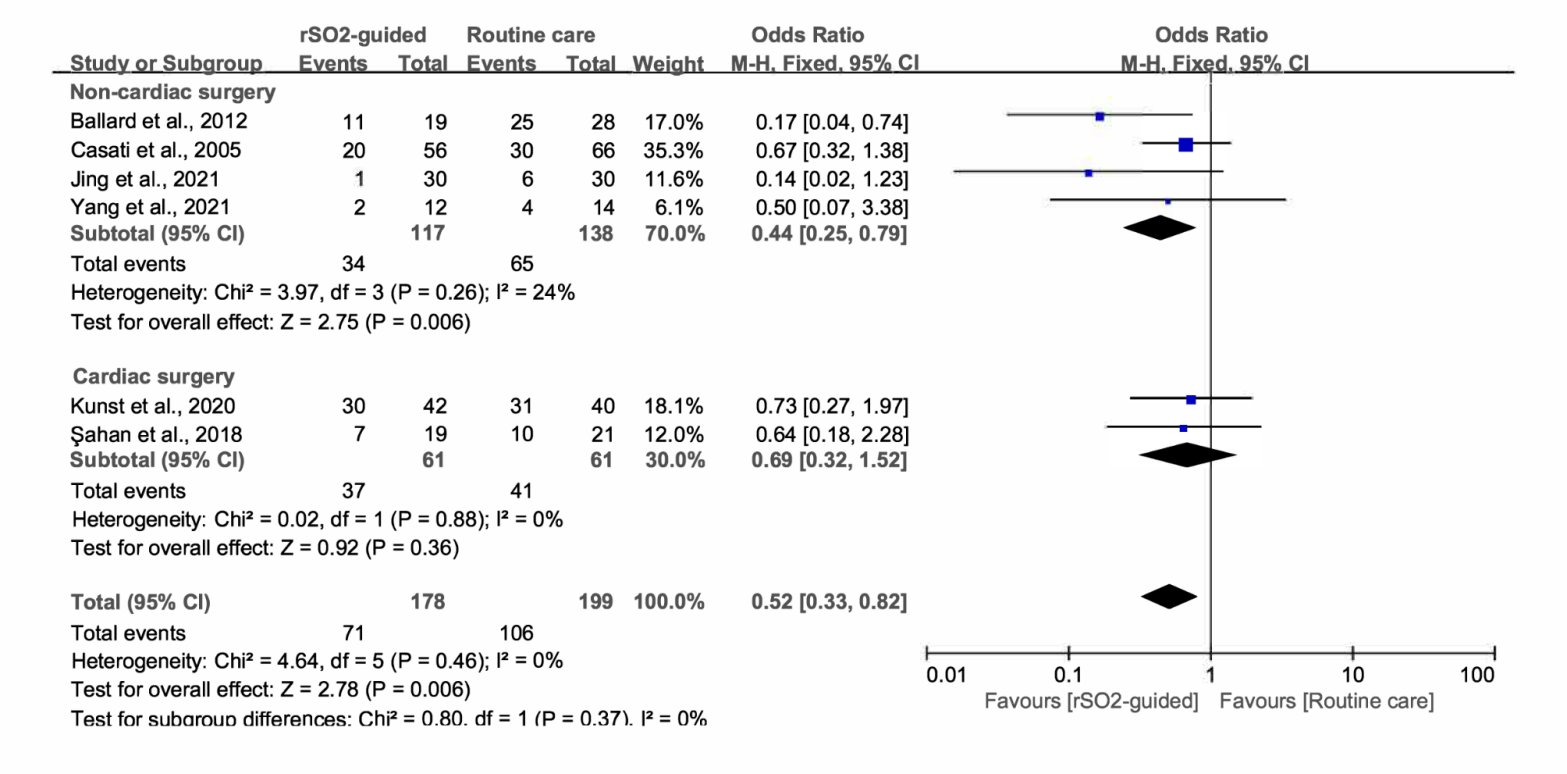
Forest plot of the subgroup analysis illustrating the incidence of POCD based on different types of surgery

After pooling and analyzing the data from the six RCTs, POCD was found to occur with an overall incidence of 46.95% (rSO_2_-guided, 39.89%; routine care, 53.27%). In general, there was a significant decrease in the incidence of POCD in the rSO_2_-guided group compared to the routine care group (OR, 0.52; 95% CI, 0.33 to 0.82; *P* = 0.006) without heterogeneity (*I*^*2*^ = 0, *P* = 0.46) (Fig. 3). In the subgroup analysis, studies involving non-cardiac surgery showed a similar association (OR, 0.44; 95% CI, 0.25 to 0.79; *P* = 0.006; *I*^*2*^ = 24), but we found no significant association in cardiac surgery (OR, 0.69; 95% CI, 0.32 to 1.52; *P* = 0.36; *I*^*2*^ = 0) (Fig. 4).

### Postoperative complications

**Fig. 5:**
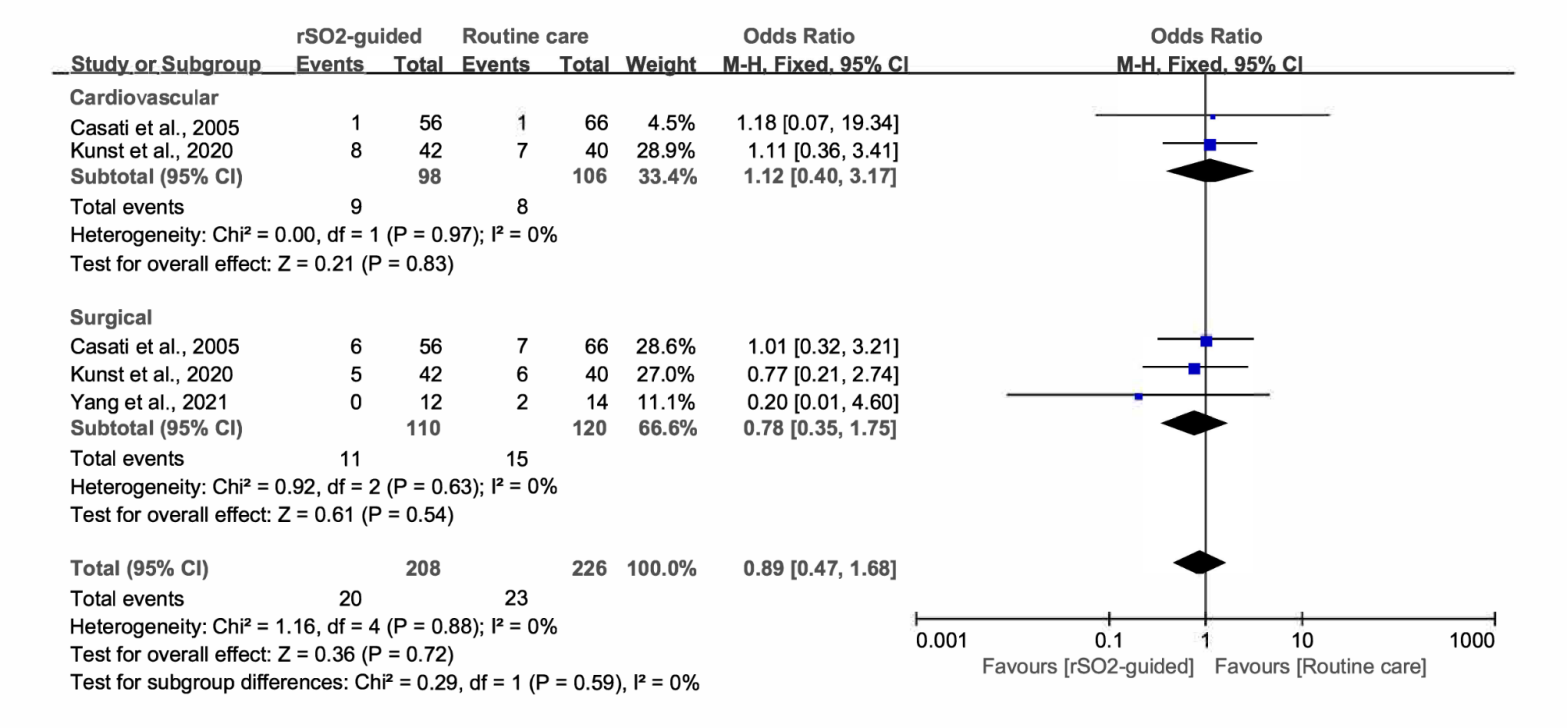
Forest plot illustrating the incidence of postoperative cardiovascular and surgical complications between intervention group (rSO_2_-guided anesthesia) and control group (routine care)

Two RCTs specifically assessed the occurrence of postoperative cardiovascular complications [27, 29]. Kunst et al. [29] defined them as new-onset atrial fibrillation requiring medical treatment. However, Casati et al. [27] did not identify any complications. The results of the pooled analysis suggested no significant difference between the two groups (OR, 1.12; 95% CI, 0.40 to 3.17; *P* = 0.83; *I*^*2*^ = 0) (Fig. 5).

Three RCTs compared surgical complications between the groups, such as postoperative infection or fever [7, 27, 29]. We also found no statistically significant difference between the rSO_2_-guided and routine care groups (OR, 0.78; 95% CI, 0.35 to 1.75; *P* = 0.54; *I*^*2*^ = 0) (Fig. 5).

### Length of hospital stay (LOS)

**Fig. 6:**
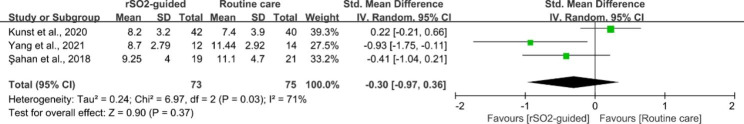
Forest plot illustrating the length of hospital stay between intervention group (rSO2-guided anesthesia) and control group (routine care)

**Fig. 7:**
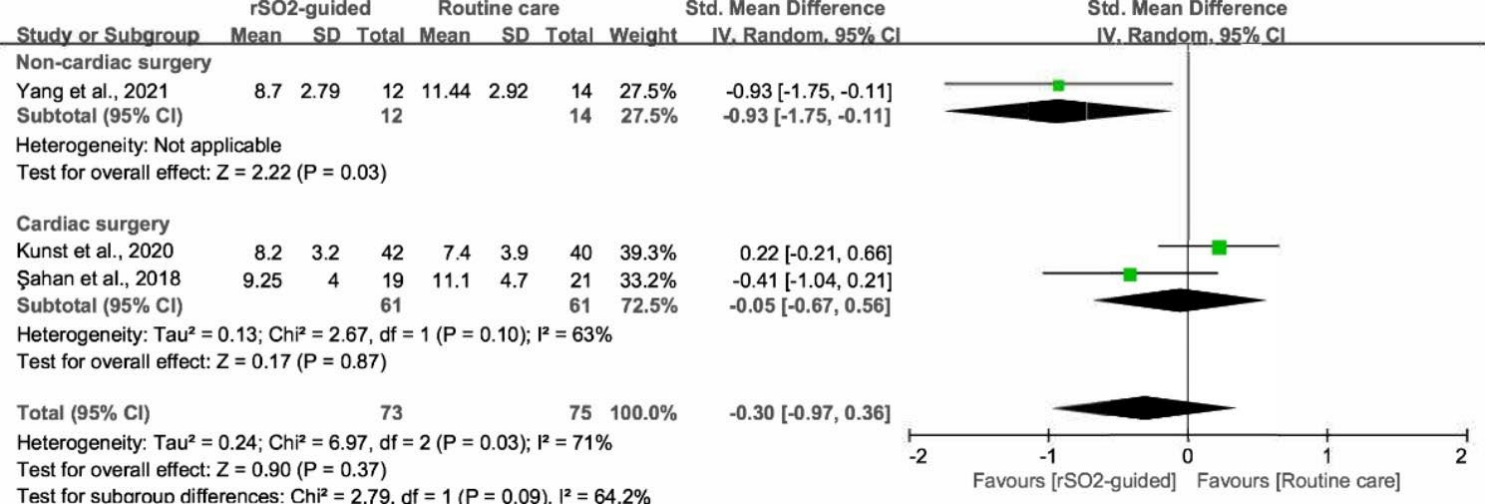
Forest plot of the subgroup analysis illustrating the length of hospital stay based on different types of surgery

LOS was examined in three RCTs, including two studies on cardiac surgery [29, 30] and one study on non-cardiac surgery [7]. In general, our results suggest that there was no difference in LOS between the two groups (SMD, -0.30; 95% CI, -0.97, 0.36; *P* = 0.37; *I*^*2*^ = 71%) (Fig. 6). We then conducted a subgroup analysis according to the type of surgery, which demonstrated that the use of rSO_2_ monitoring decreased LOS in the non-cardiac surgery subgroup (SMD, -0.93; 95% CI, -1.75 to -0.11; *P* = 0.03), but not in the cardiac surgery subgroup (SMD, -0.05; 95% CI, -0.67, 0.56; *P* = 0.87) (Fig. 7).

### Heterogeneity and publication bias

**Fig. 8:**
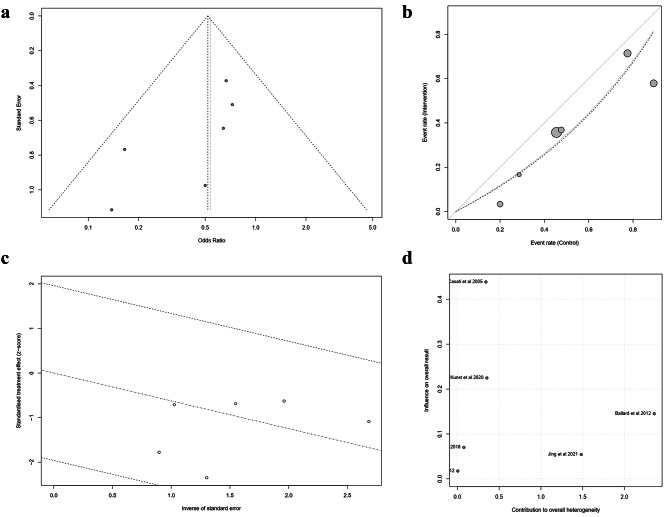
Heterogeneity and publication bias. **(a)** Funnel plot illustrating the publication bias and the systematic heterogeneity of the included studies; **(b)** L’Abbe plot illustrating the heterogeneity of the included studies; **(c)** Galbraith plot illustrating the contribution of individual studies to the heterogeneity metrics and identify outliers; **(d)** Baujat plot depicting the contribution of individual studies to overall heterogeneity

The funnel plot analyzing the publication bias in all included studies is shown in Fig. 8a, which presents a visually symmetrical distribution. Egger’s test also indicated that no significant difference was observed in publication bias (t = -2.07, *P* = 0.107). However, the results of such analyses should be treated with considerable caution, owing to the small number of studies. In addition, the L’Abbe, Galbraith, and Baujat plots of the six RCTs all suggested that there was no possible heterogeneity among these studies (Fig. 8b and d).

## Discussion

To our knowledge, this is the first meta-analysis to specifically explore the effects of rSO_2_ monitoring on the occurrence of POCD and other adverse postoperative complications in older patients, which was assessed based on data from 377 patients from six RCTs. The incidence of POCD ranges from 17 to 89%, with an overall prevalence of 47% in this meta-analysis. The results of our analysis demonstrated that rSO_2_-guided intervention could remarkably decrease the incidence of POCD and shorten LOS among older patients undergoing non-cardiac surgery. However, our pooled results did not show that the incidence of postoperative cardiovascular or surgical complications was affected by the use of intraoperative cerebral oximetry.

POCD is characterized by a deterioration in cognitive performance after surgery, which is particularly prevalent in older patients. To date, there is no consensus on neuropsychological tests specifically used for POCD [31]. It is usually detected with different meticulous neuropsychological tests, such as MMSE, MoCA, and neuropsychological test battery, which were all cited in our present meta-analysis [32]. These cognitive tests present different sensitivity, specificity, test duration and covered domains [33]. The substantial heterogeneity in methodology subsequently limits comparability and affects consistency of findings. MMSE is a commonly used test for POCD, and a follow-up measurement within seven days postoperatively seems to be broadly accepted [34]. Notably, however, MMSE lacks the sensitivity and specificity in capturing subtle cognitive deficits [31, 33]. Compared with MMSE, neuropsychological test batteries are more sensitive and specific but often complicated and time consuming. Furthermore, these test batteries are often delivered by trained staffs, so they are difficult to be popularized and applied in perioperative settings [35]. We believe that strong efforts are necessary to explore precise and applicable assessment methods for POCD.

POCD is more frequent and lasts longer in older patients following surgery under anesthesia, which may be mainly due to degenerative changes in the structure of the brain and a progressive decline in reserve function [36]. It is generally accepted that advanced age, especially pre-existing cognitive impairment, is associated with a high incidence of POCD [37, 38]. Episodes of cerebral ischemia and hypoxia have been regarded as the most closely related to POCD among distinct etiological factors [39–41]. Compared with younger patients, older patients are more predisposed to perioperative ischemia-induced brain injury, which is partly due to their reduced physiologic cerebrovascular reserve induced by atherosclerosis, hypertension, diabetes, smoking, etc. [42, 43]. Moreover, brain white matter lesions, which are frequently produced by chronic cerebral hypoperfusion in the older population, have been demonstrated to exacerbate the risk of POCD [44]. The basic value of rSO_2_ is lower in older patients [45]. In summary, poorer cognitive outcomes following surgery under anesthesia might be a consequence of more frequent and severe cerebral hypoxemia and hypoperfusion in older patients.

As a continuous and non-invasive technology, NIRS can penetrate the brain at a depth of 3–4 cm below the skin and estimate oxygenation in detected brain tissue [12, 46, 47]. Several trials have demonstrated a close association between perioperative rSO_2_ value and postoperative cognitive outcomes in older patients, which may offer a unique opportunity to elucidate the neuropathological mechanisms of POCD [13, 28, 48]. We arrived at a similar conclusion in the present meta-analysis. However, other studies failed to reveal such an association [29, 30]. Zorrilla-Vaca et al. included 15 RCTs comprising 2,057 patients in a meta-analysis to estimate the effects of intraoperative rSO_2_-based management on clinical outcomes, which suggested that the use of rSO_2_ monitoring was related to a reduction in the occurrence of POCD, but the heterogeneity within the included studies was high [49]. This result was similar to those reported by Ding et al. [19] and Chen et al. [20]. However, the findings of a Cochrane review suggested that the effects of rSO_2_ monitoring on POCD were uncertain owing to the low quality of evidence and high heterogeneity among the included studies [22]. We speculated that the inconsistent results were probably due to the principles of rSO_2_ monitoring and differences among the included participants. On the one hand, rSO_2_ measurement may interfere with the increased distance between the skin and brain tissue, such as in the case of cortical atrophy in older patients. On the other hand, the rSO_2_ value reflects mixed arterial and venous saturation in localized areas of the frontal lobes, but not the whole brain. Our meta-analysis showed that rSO_2_-guided intervention could reduce the incidence of POCD in older patients undergoing non-cardiac surgery rather than cardiac surgery. Cardiac surgery with cardiopulmonary bypass has been shown to induce microthromboembolic event-related cerebral microvascular dysfunction [50–52]. If cerebral microemboli do not occur in the frontal cortex, false-negative NIRS results will be recorded, which means that intraoperative rSO_2_ values may remain normal in cases of severe cerebral ischemia in other brain regions. The findings of Rummel et al. support our hypothesis, which suggests that the rSO_2_ value remains normal even in severe hemispheric stroke because the anterior cerebral artery can be supplied by the contralateral side [53]. In non-cardiac surgery, systemic hypotension or anemia may be responsible for a reduction in global cerebral blood flow and oxygen supply, which can be effectively reflected by cerebral desaturation in the frontal lobe in older patients. Therefore, using NIRS to manage anesthesia during major non-cardiac surgery may help alleviate global cerebral ischemia and hypoxia and decrease the risk of POCD.

LOS, an important and practical indicator, is commonly used to assess overall healthcare utilization. In the present meta-analysis, we found that the LOS of older non-cardiac surgical patients who did not receive intraoperative rSO_2_ monitoring was significantly prolonged and was accompanied by an increased incidence of POCD. A recently published prospective study revealed that older surgical patients with POCD are prone to need a prolonged LOS, which suggests the potential consequence of POCD, although clinically subtle, has a noticeable adverse impact on healthcare system expenditure [10]. Therefore, we should actively apply appropriate strategies, such as intraoperative rSO_2_ monitoring, to prevent the occurrence of POCD in a high-risk population. Meanwhile, there may be one possible cause for our failure to show a prophylactic effect of intraoperative rSO_2_ monitoring against postoperative cardiovascular or surgical complications in older surgical patients. Compared to the brain, other vital organs and surgical incisions are more tolerant to ischemia/hypoxia-induced injury. When cerebral desaturation leads to neurological damage, other tissues may not suffer from ischemia/hypoxia-related dysfunctions.

The current meta-analysis has several potential limitations. First, the population we focused on was patients older than 60 years, which may limit the generalizability of the results. Second, only two literatures on cardiac surgery were included in our meta-analysis, which makes the analysis less convincing. The present study provided preliminary results owing to the small sample size, which requires further large RCTs to clarify the neuroprotective effects of rSO_2_ in older surgical patients. Third, diverse neuropsychological tests were applied, including MMSE and MoCA, which also influenced our results. Finally, well-defined reference rSO_2_ values and clinically relevant thresholds for cerebral desaturation must be explored and subsequently established in future studies.

## Conclusion

In summary, we demonstrated that rSO_2_-guided interventions can remarkably decrease the incidence of POCD in older surgical patients. Moreover, rSO_2_ monitoring is associated with a lower risk of POCD and significantly shorter LOS in older patients undergoing non-cardiac surgery. These results should be interpreted with caution, and additional prospective research is needed. As a potentially useful monitoring tool, rSO_2_ monitoring may have a reasonable prospect for predicting and preventing POCD in a high-risk population.

## Electronic supplementary material

Below is the link to the electronic supplementary material.

Supplementary Material 1

Supplementary Material 2

## Data Availability

All related data materials have provided in the supplementary material and we have cited all eligible included studies.

## Abbreviations

CABG: coronary artery bypass graft
CDT: clock drawing test
CG: control group
CI: confidence interval
DSS: digit span subtest
FiO_2_: fractional percentage of inspired oxygen
GRADE: Grades of Recommendation, Assessment, Development, and Evaluation
IG: intervention group
LOS: length of hospital stay
MMSE: Mini-Mental State Examination
MoCA: Montreal Cognitive Assessment
NIRS: near-infrared spectroscopy
OR: odd ratio
PaCO_2_: pressure of arterial carbon dioxide
POCD: postoperative cognitive dysfunction
PROSPERO: Prospective Register of Systematic Reviews
RCTs: randomized controlled trials
rSO_2_: regional cerebral oxygen saturation
SMD: standardized mean difference
VSST: visuo-spatial skills test
WLGT: word list generation test
WMS: Wechler memory scale
