# Supplementary material for: Effects of regional cerebral oxygen saturation monitoring on postoperative cognitive dysfunction in older patients: a systematic review and meta-analysis
Source: BMC Geriatr. 2023 Mar 6;23:123. doi: 10.1186/s12877-023-03804-6 (PMC9987102; doi:10.1186/s12877-023-03804-6)
Supplement: Supplementary file 2 — Supplementary Material 2 [file 12877_2023_3804_MOESM2_ESM.docx]

**Literature search strategy**

**Appendix 1.** Search strategy for PubMed

((POCD) OR (postoperative cogniti*) OR (post-operative cogniti*) OR (postoperative neurocogniti*) OR (post-operative neurocogniti*) OR (PND) OR (perioperative neurocogniti*) OR (peri-operative neurocogniti*)) AND ((rSO2) OR (cerebral oximetry) OR (cerebral oxygen*) OR (cerebral regional oxygen*) OR (regional oxygen*) OR (regional tissue oxygen*) OR (oxygen saturation*) OR (NIRS) OR (near infrared spectroscopy) OR (near-infrared spectroscopy) OR (near infra-red spectroscopy)) AND ((aged*) OR (old*) OR (ageing*) OR (geriatric*) OR (elder*))

**Appendix 2.** Search strategy for Web of Science

TS=(POCD OR postoperative cogniti* OR post-operative cogniti* OR postoperative neurocogniti* OR post-operative neurocogniti* OR PND OR perioperative neurocogniti* OR peri-operative neurocogniti*) AND TS=(rSO2 OR cerebral oximetry OR cerebral oxygen* OR cerebral regional oxygen* OR regional oxygen* OR regional tissue oxygen* OR oxygen saturation* OR NIRS OR “near” infrared spectroscopy OR near-infrared spectroscopy OR “near” infra-red spectroscopy) AND TS=(aged* OR old* OR ageing* OR geriatric* OR elder*)

**Appendix 3.** Search strategy for Embase

#1

'POCD':ab,ti OR 'postoperative cogniti*':ab,ti OR 'post-operative cogniti*':ab,ti OR 'postoperative neurocogniti*':ab,ti OR 'post-operative neurocogniti*':ab,ti OR 'PND':ab,ti OR 'perioperative neurocogniti*':ab,ti OR 'peri-operative neurocogniti*':ab,ti

#2

'rSO2':ab,ti OR 'cerebral oximetry':ab,ti OR 'cerebral oxygen*':ab,ti OR 'cerebral regional oxygen*':ab,ti OR 'regional oxygen*':ab,ti OR 'regional tissue oxygen*':ab,ti OR 'oxygen saturation*':ab,ti OR 'nirs':ab,ti OR 'near infrared spectroscopy':ab,ti OR 'near-infrared spectroscopy':ab,ti OR 'near infra-red spectroscopy':ab,ti

# 3

'aged*':ab,ti OR 'old*':ab,ti OR 'ageing*':ab,ti OR 'geriatric*':ab,ti OR 'elder*':ab,ti

# 4

#1 AND #2 AND #3

**Appendix 4.** Search strategy for Cochrane Library

#1

"POCD":ti,ab,kw OR "postoperative cogniti*":ti,ab,kw OR "post-operative cogniti*":ti,ab,kw OR "postoperative neurocogniti*":ti,ab,kw OR "post-operative neurocogniti*":ti,ab,kw OR "PND":ti,ab,kw OR " perioperative neurocogniti*":ti,ab,kw OR " peri-operative neurocogniti*":ti,ab,kw

#2

"rSO2":ti,ab,kw OR "cerebral oximetry":ti,ab,kw OR "cerebral oxygen*":ti,ab,kw OR "cerebral regional oxygen*":ti,ab,kw OR "regional oxygen*":ti,ab,kw OR "regional tissue oxygen*":ti,ab,kw OR "oxygen saturation*":ti,ab,kw OR "NIRS":ti,ab,kw OR "near infrared spectroscopy":ti,ab,kw OR "near-infrared spectroscopy":ti,ab,kw OR "near infra-red spectroscopy":ti,ab,kw

# 3

"aged*":ti,ab,kw OR "old*":ti,ab,kw OR "ageing*":ti,ab,kw OR "geriatric*":ti,ab,kw OR "elder*":ti,ab,kw

# 4

#1 AND #2 AND #3
